# Silico-tuberculosis and associated risk factors in central province of Iran

**DOI:** 10.11604/pamj.2015.20.333.4993

**Published:** 2015-04-07

**Authors:** Aliasghar Farazi, Mansooreh Jabbariasl

**Affiliations:** 1 Department of Infectious Diseases, School of Medicine, Arak University of Medical Sciences, Arak, Iran; 2 Department of Disease Control and Prevention, Health Center of Markazi Province, Arak University of Medical Sciences, Arak, Iran

**Keywords:** Prevalence, silicosis, tuberculosis

## Abstract

**Introduction:**

Co-existence of silicosis and tuberculosis is known as silico-tuberculosis. This article review the frequency of silicosis and tuberculosis in workers who exposed to silica and evaluate influencing factors that may increase the risk of silico-tuberculosis.

**Methods:**

An analytical cross-sectional study was performed in silica exposed workers in central province of Iran during 2011-2012. Sampling method was un-randomized and considering all workers who at least 6 months exposed to silica. The study was done via questionnaire, clinical examination, spirometry, chest x-ray and tuberculosis investigations.

**Results:**

A total of 3121 workers were included in the study, the mean age of participants was 43.1±12.4 years, and mean employment duration 14.9±6.8 years. Prevalence of TB in silica-exposed workers without silicosis was 172 cases per 100 000 people and prevalence in silicosis cases was 917 cases per 100 000 people. Incidence of TB in silica-exposed workers without silicosis was 69 cases per 100 000 people and incidence in silicosis cases was 459 cases per 100 000 people. The frequency of LTBI/TB was higher in age over thirty years old (P = 0.02), in workers with employment duration over 10 years (P = 0.004), in workers with exposure duration over 5 years (P = 0.03) and smokers with over 5 pack-years (P = 0.01).

**Conclusion:**

Exposure to silica causes a renewed multiplication of bacilli in the healing TB lesions. Prevalence of pulmonary tuberculosis in Silicosis is more common when compared to prevalence in general population, hence all should use prophylactic measures Intensification of work place.

## Introduction

Silicosis is a chronic lung disease caused by breathing of silica dust. Silica is one of the common mineral in the earth′s crust. People who work in jobs where they exposed to silica bits are at risk for silicosis. Silicosis or even exposure to silica without established disease is associated with increased risk of developing various comorbidities. The World Health Organization (WHO) reported that the incidence rate of TB were 128 cases per 100 000 population worldwide in 2010 [1]. As a result of improvement in population health, and global Stop TB Strategy, the number of annual incident cases has been falling since 2006. Similarly, the incidence rate has been decreasing since year of 2002 [2, 3]. About one-eighth of incident cases were co-infected with HIV that 82% of whom were in the African Region of the WHO in year of 2010. Furthermore, there were an estimated 1.4 million people who died as a result of TB in 2010, 25% of whom were co infected with HIV [4]. Tuberculosis (TB) is one of the most important infectious diseases in the world in terms of morbidity and mortality and in year of 2012 its prevalence and incidence rate in Iran were 33(13-61) and 21(15-28) per 100 000 population respectively [5]. Among patients with silicosis higher prevalence of tuberculosis and non-tuberculosis mycobacteria have been documented [6]. In resource-limited countries relatively little is known of the burden of lung disease among exposed workers owing to a lack of surveillance and poor access to health services. The risk of a patient with silicosis developing pulmonary tuberculosis is reported to be 2.8 to 39 times and for extra-pulmonary tuberculosis is also as much as 3.7 times higher than in healthy population [7]. The pleural form is most common, accounting for 61% of the cases, followed by the pericardial form and the lymph node form [8]. In a prospective study in 2000 miners, showed that the risk of developing pulmonary tuberculosis is proportional to the severity of the silicosis and the intensity of the exposure and workers with the highest cumulative exposure to dust were 3.2 times more likely to develop tuberculosis than were those with the lowest loads. In another study observed a mean duration of 6.8 years between the diagnosis of silicosis and the onset of tuberculosis [9].

Experimental studies showed that silica impairs the function of alveolar macrophages, and severe exposure causes macrophage apoptosis. These findings are consistent with observations that the incidence of tuberculosis in dust-exposed workers is higher even in those without established silicosis [9]. Another element involved is surfactant protein A, and excess of this protein seems to be associated with higher susceptibility to tuberculosis, possibly because it inhibits the formation of reactive nitrogen species by the activated macrophages and allows mycobacteria to enter the alveolar macrophages without triggering cytotoxicity [10]. It is also believed that the bacilli can remain encapsulated within the silicosis nodules, which would be responsible for the reactivation of tuberculosis in such patients. This study was performed for assessment of silicosis and silico-tuberculosis among silica exposed workers and focus on the association between silicosis and the development of tuberculosis, evaluate influencing factors that may increase the risk of silico-tuberculosis for prevention and control of disease by surveillance and early detection.

## Methods

The present analytical cross-sectional study evaluated all workers (3700 males) who worked in mining, mineral processing, stone cutting, pottery and glass manufacturing, Masonry work, Rock drilling, Sand and gravel screening in Markazi province between 2011 and 2012. Of 3700 male workers who enrolled, 3185 (86%) workers completed the questionnaire and tuberculin skin test (TST) results were recorded correctly. Data on smoking habits, radiography, spirometry and physical examination were incomplete in 64 workers so analyses were performed on 3121 workers. The study protocol was approved by the Research Ethics Committee at the Arak University of Medical sciences. After getting informed consent, data collection was done according to clinical examination forms and a respiratory status checklist (approved by the Faculty of Health at the Arak University of Medical sciences) through interviewing and clinical examination. Spirometry for assessment of lung volumes and capacities and tuberculin skin test was performed for all cases at the same time and if the TST was negative, it was performed again within two weeks for confirmation. All study participants were referred to a radiologist in to obtain chest radiography and based on radiography, TST results and physical examination overnight collection of sputum was examined for presence of acid fast bacilli by smear (Ziehl-Neelsen stain) for 3 consecutive days and culture were done on LJ Medium & incubated up to 8 weeks before reporting negative. Afterwards, spirometry results, chest radiographies, Tuberculin test, sputum smear results, and physical examination were evaluated by specialists and according to WHO (World Health Organization), ATS (American Thoracic Society) and ILO (International Labor Organization) guidelines, silicosis, tuberculosis and silico-tuberculosis were confirmed [11, 12]. Workers were classified into four possible outcomes of the spectrum of TB infection and disease. 1) LTBI (Latent Tuberculosis Infection) defined by positive results for PPD, Chest radiograph is typically normal, no sign of active TB, negative smear and culture results; 2) No LTBI and no TB defined as negative PPD results, chest radiographs showing no sign of previous or active TB disease, and negative smear and culture results; 3) Past TB defined as positive PPD results, chest radiographs showing signs of previous TB, negative smear and culture results; 4) Active TB defined as chest radiographs showing signs of active TB and/or positive smear results and/or positive culture results.

Collected data were analyzed using SPSS software (version 18). Statistical Analysis were performed by calculation of mean, standard deviation and significance of proportional differences between nominal variables was determined using the Fisher′s exact test or chi-square test, and differences between continuous variables were determined using student t-test. Logistic regression analysis was used in order to control for confounding factors and assess the correlation of silica exposure with TB. Also, Odds ratio (OR) with 95% confidence interval (95%CI) was applied to evaluate the effect of silica exposure and different factors on development of TB. A two-tailed p < 0.05 was used to define statistical significance.

## Results

A total of 3121 workers were included in the study, the mean age of participants was 43.1±12.4 years (range 16 to 66) and median was 38 years, and mean employment duration 14.9±6.8 years (range 2 to 28). Current smoking rate was 38%, with ever smoking 57% and 15% were addicted to Opium. Among our participants, 1635(52.4%) cases had positive tuberculin test (induration ≥ 10 mm) and 218(7%) workers had silicosis, as well as 3 (0.10%) active pulmonary tuberculosis (TB) cases and 4 (0.13%) past tuberculosis cases (3 pulmonary and 1 extra-pulmonary TB) were diagnosed. Among silicosis cases, 166 (76.2%) workers had exposure for more than five years and 98(45%) workers were smokers, and 1(0.5%) worker had active tuberculosis and 1(0.5%) workers had past tuberculosis. Forty two percent of workers in our study was aware of safety measures regarding protection from silica dust in working area. In silicosis cases cough with expectoration comprised of the 129 (59.2%), followed by dyspnea 114 (52.3%), chest pain 86 (39.5%), clubbing 67 (30.7%), hemoptysis 19 (8.7%), lymphadenopathy 11 (5.1%) and hoarseness of voice 6 (2.8%). Prevalence of TB in silica-exposed workers without silicosis was 172 cases per 100 000 people and prevalence in silicosis cases was 917 cases per 100 000 people. Incidence of TB in silica-exposed workers without silicosis was 69 cases per 100 000 people and incidence in silicosis cases was 459 cases per 100 000 people. Demographic and background characteristics in silicosis and non-silicosis workers are shown in Table 1. The frequency of LTBI/TB was higher in age over thirty years old (P = 0.02), in workers with employment duration over 10 years (P = 0.004), in workers with exposure duration over 5 years (P = 0.03) and smokers with over 5 pack-years (P = 0.01) (Table 2).

**Table 1:** Demographic and background characteristics in silicosis and non-silicosis workers

Group Variable	Silicosis workers 218(7%)	Non-Silicosis workers 2903(93%)	P-value
Age years (Mean±SD)	45.1±12.7	42.9±11.8	0.02
Afghan (%)	23(10.6%)	409(14.1%)	0.15
Mean employment duration (Mean ±SD)	16.1±7.2	14.8±6.9	0.01
Mean exposure duration (Mean±SD)	15.8±7.5	14.2±7.1	0.003
Smoking (mean pack-years) (Mean±SD)	11.7±6.5	10.4±5.7	0.004
Opium addiction (%)	35(16.1%)	433(14.9%)	0.63
NO LTBI/TB (%)	84(38.5%)	1402(48.3%)	0.005
LTBI (%)	132(70.2%)	1496(35.6%)	0.01
Past TB (%)	1(0.5%)	3(0.1%)	0.14
Active TB (%)	1(0.5%)	2(0.07%)	0.04

**Table 2:** Logistic regression analyses of risk factors for LTBI/TB in silicosis workers

Group Variable	LTBI/TB 134(61.5%)	NO LTBI/TB 84(38.5%)	Adjusted OR (95%CI)	P-value
**Age(ys)**			1.97 (1.12-3.46)	0.02
≤30	41(30.6)	39(46.4)		
>30	93(69.4)	45(53.6)		
**Nationality**			0.65 (0.27-1.56)	0.37
Iranian	122(91.1)	73(86.9)		
Afghan	12(8.9)	11(13.1)		
**Smoking(pack-years)**			3.11 (1.30-7.44)	0.01
≤5	16(11.9)	18(21.4)		
>5	47(35.1)	17(20.2)		
**employment duration(ys)**			2.32 (1.32-4.10)	0.004
≤10	39(29.1)	41(48.8)		
>10	95(70.9)	43(51.2)		
**exposure duration(ys)**			2.07 (1.10-3.88)	0.03
≤5ys	25(18.7)	27(32.1)		
>5	109(81.3)	57(67.9)		
**Opium addiction(ys)**			0.45 (0.12-1.76)	0.32
≤5	7(5.2)	9(10.7)		
>5	12(8.9)	7(8.3)		

## Discussion

TB is among the most prevalent public health dilemmas of the 21st century [13]. Despite the vast efforts made for control and eradication of this disease, TB control programs have not been very successful in communities with high exposure to silica, and TB-related morbidity and mortality are increasing in such areas [14]. Silicosis is a well-known fibrogenic lung disease which is probably the most ancient occupational illness. LTBI is important to identify, although the lifetime risk for most individuals of disease progression to active TB is low about five to 10 percent [15]. Tuberculosis as a complication of silicosis has been a historical focus of attention over the last centuries [16]. In the current study, frequency of LTBI/TB was significantly higher in subjects with silicosis workers compared to the non-silicosis group (P = 0.005). Prevalence of TB in silica-exposed workers without silicosis was 5.2 fold and prevalence in silicosis workers was 27.8 fold higher than general population. Incidence of TB in silica-exposed workers without silicosis was 3.3 fold and incidence in silicosis cases was 21.8 fold higher than general population. In silicosis worker frequency of LTBI/TB was higher in workers with age over thirty years old, in workers with employment duration over 10 years, in workers with exposure duration over 5 years and workers with over 5 pack-years smoking, but, no such correlation was noted in workers with opium addiction or workers nationality.

In patients with silicosis, it is extremely important to exclude the coexistence of active tuberculosis. However, the diagnosis of active tuberculosis superimposed on silicosis can be very difficult, because the clinical manifestations can be benign and the radiological alterations can be indistinguishable from those resulting from the preexisting silicosis [17]. Therefore, in cases of clinical suspicion of concomitant active tuberculosis, an appropriate additional investiga¬tion should be performed. In a prospective study in Brazil it was revealed that risk of progression of pulmonary tuberculosis depended on the severity of silicosis. Workers with severe silica exposure developed TB 3.22 times more than those with the lowest exposure [18]. In a study by Rosenman et al. conducted in USA, occupational risk factors responsible for TB progression were discussed. Exposure to silica was among the most important risk factors in this regard [19]. In another study in Hong Kong, it was reported that by advanced age in silica exposed subjects, the frequency of pulmonary TB in them increased [20]. Also, in a study by Chopra et al, in India, 93% of TB patients were in the age range of 21 to 55 years; among which, the majority were 46-50 years of age [21]. Other studies also show very high frequency of TB in silica-exposed workers without silicosis [8]. In study by Yarahmadi et al. conducted in Iran showed that silica exposure was prevalent among TB patients and frequency of TB increased by increased intensity of silica exposure, older age and cigarette smoking [22].

Reduced exposure to silica has a marked effect on TB control. Silica exposure can increase the risk of TB even in absence of silicosis [8]. Some researchers believe that reduced occupational exposure to silica particles is effective for reducing TB incidence. Also, decontamination of work environment from silica particles can significantly reduce number of TB cases. This method can be employed as a TB control strategy especially in communities with high prevalence of TB and silica exposure [23].

## Conclusion

Silicosis is a prevalent disease for which there is currently no specific treatment. Silica-exposed workers, with or without silicosis, are at increased risk for tuberculosis. The risk of a patient with silicosis developing tuberculosis is higher (2.8 to 39 times) than that found for healthy controls. However, since both silica dust and silicosis increase the risk of pulmonary TB, it would be a necessary to reducing this risk that should be done by regular health check-up and educational programme for all silica exposed workers and smoking should be prohibited in these workers and a good care and support for those diagnosed with these conditions. The important limitation in this study related to the lack of evaluation of type and concentration of silica particles, short length of observation, and difficulty of diagnosis silico-tuberculosis. We propose that further studies be conducted to investigate strategies contribute to silicosis and silico-tuberculosis control and prevention.
